# Synthetic lethal mutations in the cyclin A interface of human cytomegalovirus

**DOI:** 10.1371/journal.ppat.1006193

**Published:** 2017-01-27

**Authors:** Henry Weisbach, Christoph Schablowsky, Barbara Vetter, Iris Gruska, Christian Hagemeier, Lüder Wiebusch

**Affiliations:** Charité Universitätsmedizin Berlin, Labor für Pädiatrische Molekularbiologie, Berlin, Germany; University of Alabama at Birmingham, UNITED STATES

## Abstract

Generally, the antagonism between host restriction factors and viral countermeasures decides on cellular permissiveness or resistance to virus infection. Human cytomegalovirus (HCMV) has evolved an additional level of self-imposed restriction by the viral tegument protein pp150. Depending on a cyclin A-binding motif, pp150 prevents the onset of viral gene expression in the S/G2 cell cycle phase of otherwise fully permissive cells. Here we address the physiological relevance of this restriction during productive HCMV infection by employing a cyclin A-binding deficient pp150 mutant virus. One consequence of unrestricted viral gene expression in S/G2 was the induction of a G2/M arrest. G2-arrested but not mitotic cells supported viral replication. Cyclin A destabilization by the viral gene product pUL21a was required to maintain the virus-permissive G2-arrest. An HCMV double-point mutant where both pp150 and pUL21a are disabled in cyclin A interaction forced mitotic entry of the majority of infected cells, with a severe negative impact on cell viability and virus growth. Thus, pp150 and pUL21a functionally cooperate, together building a cell cycle synchronization strategy of cyclin A targeting and avoidance that is essential for productive HCMV infection.

## Author summary

Efficient virus replication depends on continuous, uninterrupted supply with metabolites and replication factors from the host cell. This is difficult to achieve in actively dividing cells, especially for a slowly replicating virus like HCMV, a widespread pathogen of major medical importance in immunocompromised patients. To ensure that viral replication is not disturbed by cell division, HCMV has developed a twofold strategy of cyclin A targeting and avoidance. First, HCMV employs the viral cyclin A substrate pp150 to synchronize the onset of replication with G1, a cell cycle phase of low cyclin A expression. Then, HCMV expresses the cyclin A destabilizing factor pUL21a to maintain the G1 cell cycle state until the successful release of virus progeny. While this strategy is based on two viral proteins, a cyclin A sensor and effector, it relies on one and the same type of cyclin A interaction motif, making HCMV vulnerable to binding site disruption.

## Introduction

Control of the cell division cycle by cyclins, cyclin-dependent kinases (CDKs) and CDK inhibitors (CKIs) is fundamental for proliferation, development and homeostasis of multicellular organisms [1, 2]. To reprogram the cell cycle for their own benefit, viral pathogens have evolved, or acquired from their host, genes and sequence motifs facilitating direct interaction with the cyclin-CDK protein network [3].

Herpesviruses are particularly well suited for multifaceted interactions with the cell cycle machinery, owing to the large coding capacity of their genomes. The repertory of herpesviral cell cycle regulators comprises on the one hand factors leading to constitutive activation of the cell cycle. This is exemplified by the β and γ-herpesviral orthologs of cyclins [4] and CDKs [5], which release CDK substrate phosphorylation from the control of cellular cyclins and CKIs [6, 7]. On the other hand, herpesviruses target cellular cyclin-CDK activity to arrest the cell cycle at stages conducive to virus replication [8]. A recent example is the UL21a gene product (pUL21a) of human cytomegalovirus (HCMV), which is required to block DNA synthesis and mitotic entry of infected cells [9, 10]. Like CKIs of the Cip/Kip family (p21, p27, p57), pUL21a contains a high affinity RXL-type cyclin binding motif but is only a poor CDK substrate [10]. In contrast to CKIs, however, pUL21a does not act as a stoichiometric inhibitor of cyclin-CDK complexes but specifically recruits cyclin A (also referred to as cyclin A2) for proteasomal degradation [9, 10].

Viral interactions with the cell cycle are not necessarily unidirectional. HCMV encodes a second RXL-type cyclin A-binding protein, pp150 (also referred to as pUL32), that is neither an activator nor an inhibitor of the cell cycle but is itself subject of cyclin A-CDK-dependent regulation [11]. PP150 enters the host cell as part of the HCMV virion and blocks *de novo* viral gene expression in a cyclin A and CDK-dependent manner [12, 13]. In fibroblasts and other permissive cell types, this mechanism restricts the onset of viral replication to the G0/G1 phase of the cell cycle where cyclin A expression is low or absent. S/G2 cells, though, do not abrogate but only delay infection as pp150-mediated repression is relieved once cells loose cyclin A protein after cell division [14, 15]. Animal CMVs, including chimpanzee CMV, the closest relative of HCMV, lack RXL sequence motifs in their pp150 homologues and accordingly initiate viral gene expression independent of the cell cycle position at the time of infection [11, 16]. Thus, it is yet unclear what function, if any, the pp150-dependent restriction serves in the context of productive HCMV infection.

Here, we show that the pp150-RXL motif, alone, is dispensable for efficient viral growth. However, genetic disruption of both pp150 and pUL21a-RXL motifs dramatically enhances the mitotic phenotype and growth defect of a pUL21a-RXL single mutant virus. Thus, the cyclin A antagonist pUL21a and the cyclin A sensor pp150 are part of a virus-host interface, that functions as a fail-safe system securing undisturbed HCMV replication under non-mitotic conditions.

## Results

**Fig 1:**
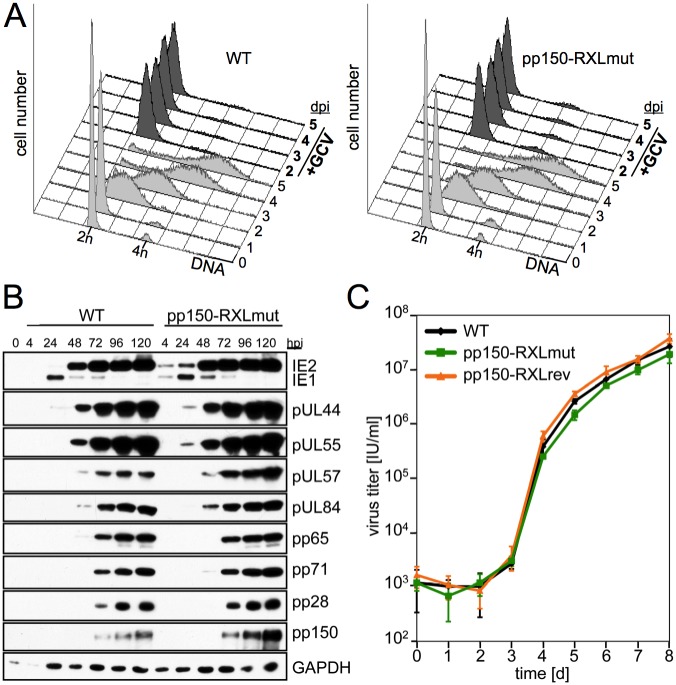
The essential function of pp150 in the late phase of HCMV infection is not compromised by lack of cylin A binding. Density-arrested fibroblasts were infected with the indicated recombinant viruses. (A) The cellular DNA content was analyzed at regular intervals by propidium iodide staining and flow cytometry. Shown are DNA histograms where a 2n (n = haploid number of chromosomes) DNA content is indicative of G0/G1 and a 4n DNA content of G2/M cells. Where indicated, ganciclovir (GCV) was added to the cells at day 1 post infection (dpi) to discriminate between viral and cellular DNA replication. (B) The expression of selected IE, early and late gene products was monitored by immunoblot analysis of whole cell lysates; loading control: GAPDH; hpi: hours post infection. (C) To obtain virus growth curves, cell culture supernatants were collected on a daily basis and analyzed for infectious titers. Data points are displayed on a logarithmic scale and represent means and standard deviations of biological triplicates.

Besides its role as a cell cycle-dependent restriction factor, pp150 has a well-documented function in the late phase of HCMV infection where it is required for capsid trafficking and stability [17–19], virion maturation and egress [20–22]. Before we began to use the cell cycle-independent HCMV-pp150-RXL mutant for investigating the consequences of unrestricted viral gene expression in S/G2, we made sure that the essential late functions of pp150 are not hampered by the cyclin binding deficiency. To this end we infected G0/G1-arrested fibroblasts, which, independent of the pp150-RXL mutation status, supported the ganciclovir-sensitive *de novo* synthesis of viral DNA (Fig 1A) and all stages of HCMV protein expression (Fig 1B). By measuring the accumulation of infectious progeny in the supernatant, we confirmed that pp150-RXL mutants were capable to grow to almost the same high levels as the corresponding WT and revertant viruses (Fig 1C). Furthermore, we made sure that the pp150-RXL mutation does not negatively affect virion infectivity (S1 Fig). Thus, in principle, pp150-RXL mutant HCMV is fully competent in virus replication and release.

**Fig 2:**
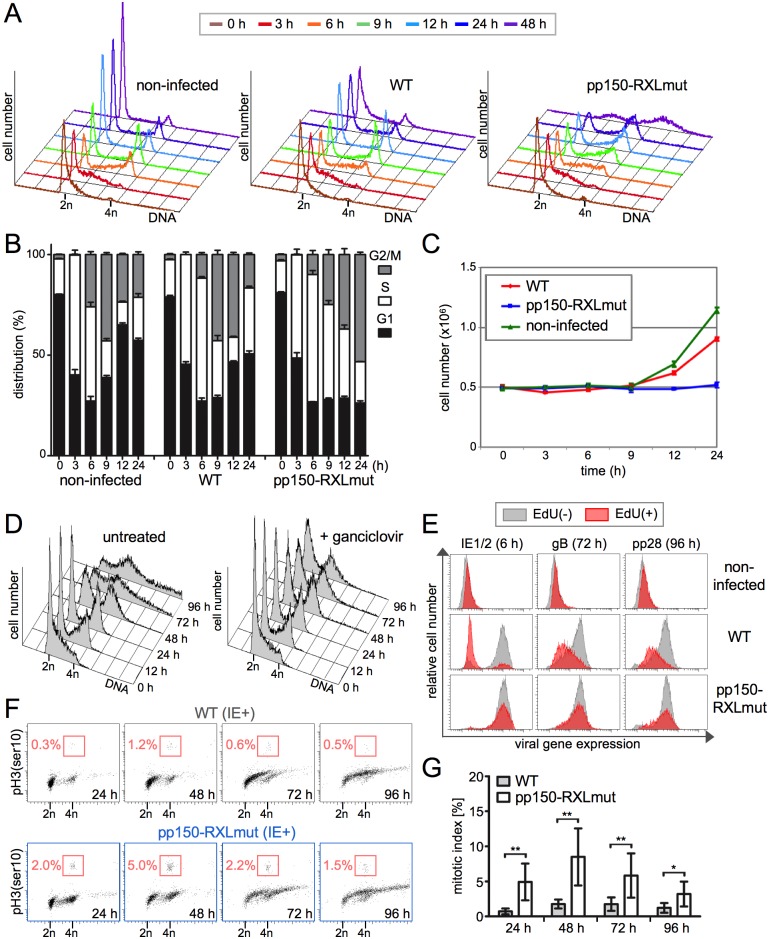
Loss of pp150-cyclin A interaction leads to an HCMV-permissive G2 arrest. Fibroblast cultures were partially synchronized in early S phase and infected with pp150-WT or pp150-RXL mutant viruses. Where indicated, cells were pulse-labeled with 5-ethynyl-2’-deoxyuridine (EdU) 60 min before infection to distinguish S phase from G1 cells. (A) Cell cycle progression of infected and non-infected control cells was analyzed by flow cytometry. (B) Distribution over G0/1, S and G2/M cell cycle phases was calculated and presented as means and standard deviations of biological triplicates. (C) To assess cell division, cell numbers were monitored over time. (D) At 24 h, HCMV-pp150-RXLmut-infected cells were either treated with ganciclovir to inhibit viral DNA synthesis or left untreated. DNA histograms were obtained by flow cytometry. (E) EdU incorporation and the expression of selected viral immediate early (IE1/2), early (gB) and late (pp28) gene products were determined by flow cytometry at different time points post infection. EdU-positive cells are displayed in red, EdU-negative cells in gray. (F) IE-positive cells were analyzed by flow cytometry for phosphorylation of histone H3 at serine 10 (pH3(ser10)), a marker of mitotic chromatin condensation. The relative proportion of mitotic cells (mitotic index) is given in percent of total cells. (G) The averages and standard deviations of mitotic indices were calculated from six independent experiments. Statistically significant differences, based on a two-tailed, paired Student’s t test, are marked with asterisks; ** (p < .01); * (p < .05).

We then infected proliferating cells, that were partially synchronized in S phase by release from contact inhibition. As expected, only pp150-RXL mutant HCMV was able to initiate viral immediate early (IE) gene expression in S phase cells (S2 Fig). Whereas HCMV-WT infected S phase cells, like non-infected control cells, were able to complete the cell cycle and divided between 6 and 24 h post infection, the pp150-RXL mutant virus blocked cell division leading to an accumulation of cells with a G2/M DNA content (Fig 2A–2C). At later times, we observed a shift of the G2/M-arrested population to a > 4n DNA content, resembling the gain of DNA content seen in the G1-arrested fractions of HCMV-WT and pp150-RXL mutant infected cells (Figs 1A and 2A). The late increase in DNA content was of viral origin as it was for the most part prevented by treatment with ganciclovir, an inhibitor of the HCMV DNA polymerase (Fig 2D). This indicated that the pp150-RXL mutant virus is able to replicate its genome in G2 cells with similar speed and efficiency as in G1 cells.

This view was further supported by analysis of viral IE, early and late gene expression. We employed a 5-ethynyl-2'-deoxyuridine (EdU) pulse labeling strategy [23] to separately track G1 and S phase-infected cells. The negative impact of S phase infection on HCMV-WT gene expression was still evident 3 to 4 days later by reduced levels of viral early (gB) and late (pp28) gene products in EdU-positive cells. In contrast, the pp150-RXL mutation allowed the cascade of lytic gene expression to proceed with similar strength and kinetics in EdU-positive and negative cells (Fig 2E). The presence of pp28 which, as a “true” late gene product, depends on viral DNA synthesis [24], supported our conclusion from the ganciclovir experiment that efficient viral replication can occur at late stages of the cell cycle, if only the cyclin A-dependent block of IE gene expression is overcome.

We then had a closer look at the cell cycle position of pp150-RXL mutant infected cells. To check whether the observed block in cell division takes place in G2 or M phase, we analyzed histone H3 serine-10 phosphorylation. Although this phosphosite has a dual role in chromosome condensation [25] and transcriptional elongation [26], and was recently found increased in HCMV-Ad169 infected interphase cells [27], the high abundance of histone H3 *de novo* phosphorylation during M phase [28] makes it a reliable and well-accepted marker of mitosis in flow cytometry and immunocytochemistry. We identified a small but, compared to WT, statistically significantly increased population of pp150-RXL mutant infected M phase cells that in contrast to G2 cells showed no signs of viral DNA replication (Fig 2F and 2G). Thus, in the absence of pp150-mediated restriction, HCMV is fully competent to replicate from the S/G2 cell cycle compartment but has an increased risk to enter into an abortive mitotic state.

**Fig 3:**
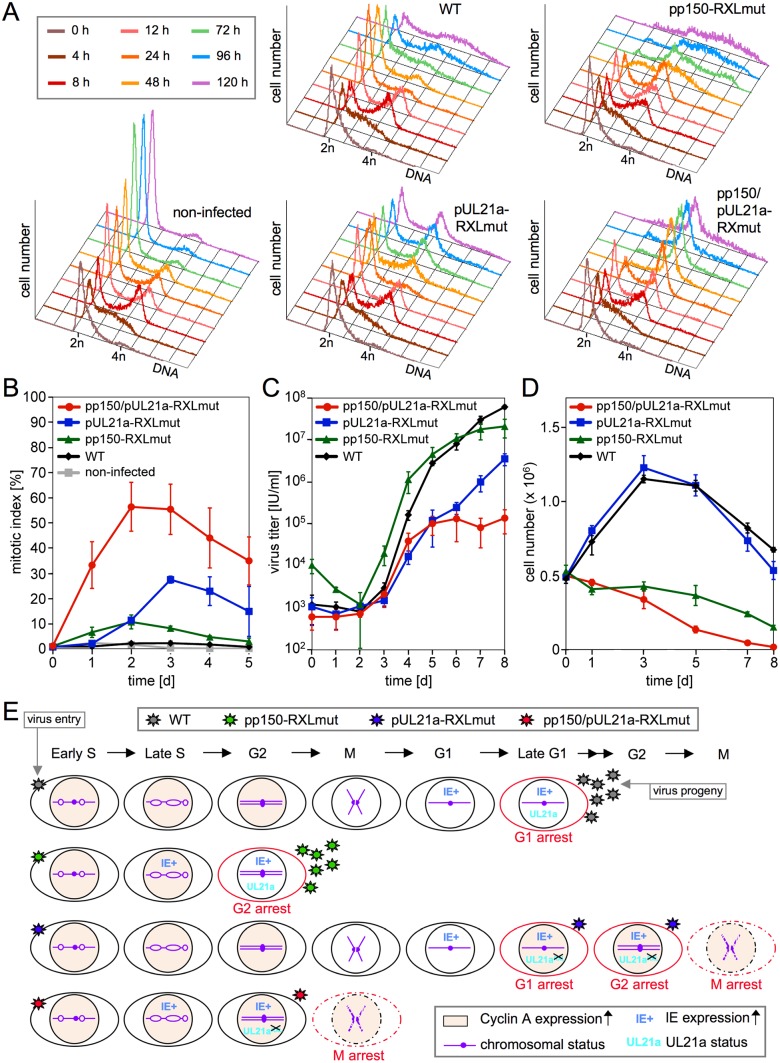
Pp150 and pUL21a cooperate to prevent HCMV-infected cells from entering a non-productive and unstable mitotic state. Fibroblasts were first synchronized in early S phase and then (at time point 0 h) infected with the indicated recombinant viruses. Cells and cell culture supernatants were harvested at regular intervals and analyzed for cell numbers, cell cycle distribution and virus growth. (A) DNA histograms of propidium iodide stained cells. (B) To determine mitotic indices of infected cells, the relative numbers of pH3(ser10) and IE1/2-positive cells were quantified by flow cytometry. Data represent the means and standard deviations of biological triplicates. (C) For virus growth curves, titers of infectious virus progeny were determined in biological triplicates. (D) The numbers of viable, propidium iodide excluding cells were determined by flow cytometry. (E) The observations were summarized into a model that visualizes the chain of events after S phase infection, leading to either a virus-permissive G1/G2 cell cycle arrest or a non-permissive mitotic state and cell death. Different numbers of asterisks, symbolizing virus progeny, indicate how efficient each virus can replicate in the particular cell cycle phases. In the case of the pUL21a-RXL mutant, it is currently unclear to what extent S phase infected cells contribute to G1, G2 and M phase arrested populations.

The finding of non-permissive mitotic cells was reminiscent of the phenotype of pUL21a-RXL mutant HCMV, which has lost the capacity to block the G1/S transition by cyclin A down-regulation and in consequence, forces up to 30% of infected cells into a non-productive and genetically unstable metaphase arrest [10]. This prompted us to ask whether both mechanisms, the pp150-cyclin A-dependent restriction of viral gene expression to G0/G1 and the pUL21a-cyclin A-dependent cell cycle block may cooperate to protect HCMV from fatal entry into mitosis. To address this question we constructed a virus carrying double-point mutations of both, the pUL21a and pp150 RXL motifs and compared the effects on cell cycle progression and virus replication side by side with the corresponding single mutants and HCMV-WT. During the first 12 to 24 h after virus entry, the pp150 status clearly dominated the phenotypic outcome of HCMV infection. The pUL21a-RXL single mutant, like WT virus, was unable to start IE gene expression in S/G2 (S3 Fig), and hence also to block cell division (Fig 3A). In contrast, the pp150/pUL21a-RXL double mutant behaved like the pp150-RXL single mutant virus in these respects. From 24 h on, the consequences of uncontrolled cyclin A expression (S4 Fig) became apparent in RXL double mutant and, with a delay of further 24 h, also in pUL21a-RXL single mutant infected cells: i) cells moved from G1 towards G2/M (Fig 3A); ii) mitotic chromosome condensation was induced (Fig 3B, S5 Fig); iii) expression of mitotic kinases cyclin B, aurora A and B was strongly up-regulated above the already increased levels in WT and pp150-RXL mutant infected cells (S4 Fig). The latter observation was consistent with our previous finding [10] that loss of pUL21a-cyclin A interaction enhances the long known stimulatory effect of HCMV on cyclin B expression [29].

Not only was the timing of mitotic entry accelerated by the simultaneous deletion of both cyclin A interaction sites, its extent was greatly elevated as well (Fig 3B, S5 Fig). The mitotic index peaked at 48 h in case of the RXL double mutant, reaching 60% of IE-positive cells. At this time, only 10–12% of the pp150 or pUL21a-RXL single mutant infected cells had entered mitosis (Fig 3B), demonstrating a synergistic pro-mitotic effect of the RXL double mutation. The high and early incidence of mitotic cells had a huge negative impact on viral replication, which was reflected by the near absence of cells accumulating with a greater than 4n DNA content (Fig 3A, S3 Fig) and by an about 500-fold growth defect (Fig 3C) of the RXL double mutant. In contrast, the pp150-RXL single mutation conferred even a growth advantage on HCMV during the first 4 to 6 days after S-phase infection, compared to WT virus. This suggests that in the presence of a functional cyclin A degradation mechanism, the fast, cyclin A-resistant onset of viral gene expression outweighs the negative consequences of a moderately increased mitotic index.

Notably, the pUL21a-RXL single mutant showed a greater lag in accumulation of infectious progeny (Fig 3A) than previously reported for G0/G1 phase infection experiments [10]. That was reflected at the protein level by reduced expression of the essential viral trans-activator IE2 and of early and late gene products (S4 Fig). The changes are most likely due to the continuous presence of cyclin A in pUL21a-RXLmut infected S phase cells (S4 Fig) which in the presence of pp150-WT is known to exert a negative effect on viral gene expression [9, 12, 30]. Analysis of early (gB) and late (pp28) protein profiles in the fraction of IE-positive S/G2 cells confirmed that, in contrast to pp150-RXLmut infection, the G2 arrested state was only semi-permissive for the pUL21a-RXL single mutant in terms of viral replication (S6 Fig). Remarkably, up to 30% of all pUL21a-RXLmut and WT infected cells remained refractory to IE gene expression for 2 days, even after re-entry into G1 phase (S3 Fig). Whereas WT closed the gap to pp150-RXL mutant infections between 2 and 4 days post infection (S3 Fig), the number of IE-deficient pUL21-RXL single mutant infected cells only slowly decreased, consistent with the delayed growth of this mutant (Fig 3A).

Although mitotic entry of infected cells was characterized by a shutdown of viral gene expression (S6 Fig), the RXL double mutant was still capable to maintain viral gene expression and replication in G2 (S4 and S6 Figs). To clarify what causes the 500-fold growth defect, we analyzed the influence of mitotic entry on the stability and viability of RXL-double mutant infected cells. In fact, those cells showed, similar to the pUL21a-RXL single mutant [10], chromosomal damage that progressively leads to a complete pulverization of the chromosomal material (S7 Fig). Furthermore, the decrease of mitotic cells seen after 2 dpi (Fig 3B) was paralleled by a large die-off of double mutant infected cells (Fig 3D, S8 Fig). Seemingly, the population of double mutant infected cells is continuously depleted of cells entering mitotic catastrophe and cell death. Regarding this instability of mitotic cells, the total number of double mutant infected cells entering mitosis during the time course of the experiment probably was greatly underestimated by flow cytometry (Fig 3B, S5 Fig), which gives only a snap-shot of the relative cell cycle distribution of viable cells.

We conclude that in the absence of the pp150/pUL21a-cyclin A interface, cyclin A and cyclin A-resistant viral gene expression together lead to the fast kinetics and high penetrance of mitotic entry seen for RXL-double mutant infected cells, with severe consequences for cell survival and virus growth (Fig 3E).

## Discussion

HCMV encodes two cyclin A interacting proteins, the cyclin A-CDK substrate pp150 and the cyclin A destabilizing module pUL21a. Here we show that both proteins act synergistically, together constituting a control circuit required for the synchronization of viral replication with the cell division cycle. The tegument protein pp150 senses the cellular cyclin A status at the beginning of infection and restricts the onset of IE gene expression to the G0/G1 phase, where cyclin A2-CDK is inactive. The early gene product pUL21a maintains the status of low cyclin A2-CDK activity by targeting cyclin A for proteasomal degradation. Thus, pp150-mediated restriction of viral gene expression in S/G2 phase has a genuine function in the productive replication cycle of HCMV instead of being merely an inevitable by-product of a silencing mechanism that contributes to establishing quiescent infection in undifferentiated cells [11].

The pp150-dependent restriction to cyclin A-negative cells appears important enough to justify a significant delay of virus gene expression and replication after infection of proliferating cells (Figs 2E and 3C). This is particularly remarkable in view of the high overall growth rates of a cyclin A sensor-less pp150-RXL mutant virus in both G1 and G2 cell cycle compartments (Figs 1C and 3C). In that respect, pp150 mutant HCMV behaves like animal CMVs, which lack a pp150-cyclin A interface and therefore can efficiently replicate in S/G2 cells [11, 16]. Taken together, this may point to a model where cyclin A sensing by pp150 alone is not absolutely required for productive HCMV infection but has been developed to strengthen and support cell cycle synchronization by pUL21a. Because pUL21a is expressed with early kinetics and therefore not available during the first hours of infection [31], a pre-synchronization step by a protein like pp150, delivered by the incoming virion, makes sense and keeps lytic gene expression in safe distance from G2/M transition, giving pUL21a time to install a stable cell cycle arrest in interphase. If this presynchronization is missing the pUL21a function is not sufficient, or is not present early enough, to tightly inhibit mitotic entry in every infected cell (Figs 2F, 2G and 3B).

The described synchronization strategy of HCMV is in striking analogy to how another human pathogen, human papilloma virus (HPV) coordinates its replication with the host cell cycle. Just as HCMV, many HPV strains encode two RXL motif containing cyclin A interactors, E1 and E1˄E4 [32, 33]. The early protein E1 is a DNA helicase that, due to its cyclin A-CDK-dependent nuclear localization, is only able to initiate viral DNA synthesis in its CDK-phosphorylated form [33, 34]. This makes sense from the viewpoint of HPV given that this small DNA virus in contrast to HCMV heavily depends on the cellular DNA replication machinery, which is only available and active at times of high cyclin A-CDK activity. The late protein E1˄E4 re-localizes cyclin A-CDK to the cytoplasm, thereby preventing the onset of mitosis [35, 36]. Thus, both HCMV and HPV have evolved two layers of cyclin A interaction. The first layer consists of cyclin A-sensitive CDK substrates that confine the start of viral replication to the most suitable cell cycle phase—G1 in case of HCMV, S phase in case of HPV. The second layer consists of potent, negative regulators of cyclin A-CDK that provide stable conditions for virus growth by arresting the cell cycle in interphase (HCMV: G1 arrest, HPV: G2 arrest). It is fascinating, that two only distantly related viruses like HCMV and HPV have developed such similar strategies to best synchronize their life cycle with that of virally favorable phases of the cell cycle for reaching highest efficacy and efficiency of viral replication.

Although HCMV, HPV and many other viruses have evolved sophisticated mechanisms to prevent mitotic entry in productively infected cells [37], it is important to note that in other contexts, such as virus entry or persistence, viruses use mitosis to their own advantage. Herpesviruses as well as papillomaviruses encode mitotic chromosome tethering factors to maintain latent viral genomes episomally in dividing cells [38–41]. Papillomaviruses and select retroviruses require the nuclear envelope breakdown in mitosis for nuclear import of viral genomes [42].

Why then mitosis presents a problem when it occurs in the middle of the productive replication cycle of HCMV? Certainly, the global shutdown of gene transcription [43] and mRNA translation [44] in mitosis would seem to be counterproductive for a virus that hijacks the host cell machinery to achieve maximum replication. Also, structural changes like the nuclear envelope breakdown are potentially harmful for the functional integrity of viral replication and assembly compartments [10]. However, if mitosis would represent only a short and transient interruption in the replication cycle of HCMV, the virus could possibly cope with such perturbations. But, HCMV, as many viruses, encode potent inhibitors of the anaphase promoting complex (APC/C) to stabilize substrates of this E3 ubiquitin ligase that play a role in viral replication [45–48]. Due to the essential function of APC/C in mitosis, productively infected cells cannot simply traverse through this cell cycle phase—they become arrested at the metaphase-anaphase transition [10]. Another aspect is that HCMV expresses DNA damaging enzymes [49, 50] and subverts cellular DNA repair [51, 52]. It is unclear, as yet, to what extent virus-induced DNA breaks contribute to the chromosomal fragmentation visible in mitotic cells (S7 Fig). However, unrepaired DNA damage and prolonged metaphase arrest are known to cause cell death by mitotic catastrophe [53, 54] and, though HCMV is a master in preventing premature cell death during interphase [55], it is evidently not equally prepared to protect productively infected cells in mitosis.

## Materials and methods

### Cells and viruses

Human embryonic lung fibroblasts (Fi301) were maintained as described previously. To synchronize them in S phase, fibroblasts were first synchronized in G1 phase by contact inhibition and then seeded at lower cell density to allow reentry into the cell cycle. Thirteen to seventeen hours after re-plating when most cells had reached early S phase, they were infected. The following recombinant viruses were used: the parental WT virus HCMV-TB40-BAC4 [56], HCMV-TB40-pUL21a-RXLmut [10], the HCMV-TB40-pp150-RXL mutant RV1659 and revertant RV1677 [11]. To obtain a HCMV-TB40-pp150/pUL21a-RXL double mutant, the pUL21a-RRL^ARA^ mutation was introduced into RV1659 by traceless BAC mutagenesis [57]. Viruses were propagated on Fi301 cells and titered by flow cytometry of IE1/IE2-positive cells as described [10]. A multiplicity of infection (MOI) of 5 to 10 IE-protein-forming units (IU) per cell was used for all experiments. To determine particle-to-IU ratios in virus stocks, virion DNA was prepared by ultracentrifugation and proteinase K/SDS treatment essentially as described [58], and quantified by real-time PCR using the UL123-specific primer pair 5’-GCCTTCCCTAAGACCACCAAT-3’ / 5’-ATTTTCTGGGCATAAGACATAATC-3’. For detection of S phase cells, cells were pulse-labelled (60 min) with 10 μM 5-ethynyl-2´-desosxyuridine (EdU) before infection. Where indicated, ganciclovir was used at a final concentration of 50 μM. Giemsa staining was performed essentially as described [10].

### Flow cytometry

To analyze DNA content, EdU incorporation, viral protein expression and histone phosphorylation, cells were harvested by trypsinization, fixed and permeabilized by incubation in 75% ethanol for at least 12 h at 0°C. Afterwards, cells were stained with specific antibodies and propidium iodide as described previously [12]. The following mouse monoclonal primary antibodies were used: anti-IE1/IE2 (clone E13, Argene), anti-IE1/IE2 (clone 8B1.2, Merck-Millipore), anti-pUL55/gB (clone CH28, Santa Cruz Biotechnology), anti-pUL99/pp28 (clone CH19, Santa Cruz Biotechnology) and anti-pH3-ser10 (clone 6G3, Cell Signaling Technology). An Alexa Fluor 488-conjugated goat anti-mouse IgG antibody (Life Technologies) served as secondary reagent. Isotype-specific antibodies were used for co-staining of IE1/IE2, pUL99/pp28 (Alexa Fluor 488-conjugated goat anti-mouse IgG2a, Life Technologies), pUL55/gB (V450-conjugated rat anti-mouse IgG1, BD Biosciences) and pH3-ser10 (Alexa Fluor 647-conjugated goat anti-mouse IgG1, Life Technologies). Alternatively, a rabbit polyclonal pH3-ser10 antibody (Cell Signaling Technology) was employed. For detection of EdU-positive cells, the Click-iT EdU Alexa Flour 647 imaging kit (Life Technologies) was used according to the manufacturer´s instructions. For cell counting and live-dead cell discrimination, cells were harvested by trypsinization and resuspended in ice-cold PBS. Immediately prior to flow cytometry, propidium iodide was added to the sample at a final concentration of 25 μg/ml. Cells were analyzed with a FACSCanto II flow cytometer (BD Biosciences) using FACSDiva, CellQuest-Pro (BD Biosciences), FlowJo (FlowJo LLC) and ModFit-LT (Verity Software House) software packages. Cellular debris, cell doublets and aggregates were gated out of analysis.

### Immunoblot analysis

Whole-cell lysates were prepared, clarified, adjusted to equal protein concentrations and further processed as previously described [13]. SDS-polyacrylamide gel electrophoresis and immunoblotting were performed according to standard protocols. The following primary antibodies were applied: anti-IE1/IE2 (clone E13, Argene), anti-pUL44 (clone CH16, Santa Cruz Biotechnology), anti-pUL55/gB (clone CH28, Santa Cruz Biotechnology), anti-pUL57 (clone CH167, Santa Cruz Biotechnology), anti-pUL83/pp65 (clone CH12, Santa Cruz Biotechnology), anti-pUL84 (clone Mab84), anti-pUL99/pp28 (clone CH19, Santa Cruz Biotechnology), anti-GAPDH (clone 6C5), anti-pUL82/pp71 (clone 2H10; a gift from Tom Shenk), anti-pUL32 (clone XP1; generously provided by Bodo Plachter). All antibodies were used at a final concentration of 1 μg/ml.

## Supporting information

S1 Fig Virion infectivity is not affected by pp150-RXLmutation. Virus stocks of HCMV-WT and HCMV-pp150-RXLmut were titrated by determining the concentration of infectious, IE1/IE2 protein forming units (IU). In addition, virion DNA was isolated from virus stocks and quantified by real-time PCR. The histogram shows relative particle to IU ratios of HCMV-WT and pp150-RXLmut, with WT set to 1.0. Data represent the means and standard deviations of technical triplicates.
(TIF)

S2 Fig In the absence of pp150-cyclin A interaction S phase-infected cells immediately start viral gene expression and arrest with a 4n DNA content. Embryonic lung fibroblasts were partially synchronized in early S phase and infected with pp150-WT or pp150-RXL mutant HCMV. Cellular DNA content and expression of immediate early gene products IE1 and IE2 were analyzed during the first 24 h post infection by flow cytometry (n = haploid number of chromosomes).
(TIF)

S3 Fig In the absence of both pp150 and pUL21a-cyclin A interaction the vast majority of infected cells accumulates with a 4n DNA content and does not show signs of viral DNA replication. Partially synchronized fibroblasts were infected near the G1/S transition with the indicated HCMV variants. Major immediate early (IE) gene expression and DNA content were analyzed by flow cytometry on a daily basis. Shown are dot plots where the cellular events are divided into 6 subpopulations. Upper left region: IE^+^/DNA content = 2n; lower left region: IE^-^/DNA content = 2n; upper middle region: IE^+^/DNA content >2n and ≤4n; lower middle region: IE^-^/DNA content >2n and ≤4n; upper right region: IE^+^/DNA content >4n; lower right region: IE^-^/DNA content >4n (n = haploid number of chromosomes). A DNA content >4n indicates viral DNA replication of G2 arrested cells.
(TIF)

S4 Fig Accelerated induction of cyclin A and mitotic kinases in RXL double mutant infected cells. G1/S fibroblasts were infected with HCMV-WT, the indicated RXL mutants or left uninfected. Whole cell lysates were prepared from 0 to 96 h post infection and analyzed by immunoblotting for protein expression of cyclins, mitotic kinases and selected immediate early, early and late gene products. In addition, histone H3-serine 10 phosphorylation, pH3(ser10), was analyzed. The conditions used for immunoblot detection of pH3(ser10) were not sensitive enough to allow a comparison of pH3(ser10) levels in non-infected and HCMV-WT infected cells. Equal protein amounts were loaded, which was controlled by analysis of GAPDH expression.
(TIF)

S5 Fig Determination of mitotic index in HCMV infected cells. Fibroblasts were synchronized and infected as described above. After harvest (here: at 72 h), cells were stained with propidium iodide and monoclonal antibodies against IE1/IE2 and pH3(ser10). The percentage of mitotic, pH3(ser10) positive cells was assessed by flow cytometry. Only the fraction of IE1/IE2-positive cells was included in the analysis.
(TIF)

S6 Fig HCMV early and late gene expression is blocked in mitosis and severely delayed in in pp150-WT S/G2 cells. Fibroblasts were infected in early S phase with HCMV-WT or the indicated RXL mutants. Cellular DNA content, mitotic marker pH3(ser10) and expression of viral immediate early (IE1/2), early (gB) and late (pp28) proteins were analyzed by flow cytometry at the indicated time points. (A) To test how efficiently the HCMV replication cycle proceeds in S/G2 versus M phase, a gating strategy was designed where the quadrant of IE-positive S/G2/M cells (Q2) was subdivided into a pH3(ser10)-positive mitotic population (R1) and a pH3(ser10)-negative S/G2 population (R2). Both populations were compared with respect to gB and pp28 protein expression. (B) Shown are histogram overlays of IE-positive mitotic and S/G2 populations. Non-infected S/G2 cells were analyzed to control for non-specific (NS) background staining.
(TIF)

S7 Fig Mitotic entry of RXL double mutant infected cells is accompanied by progressive chromosomal fragmentation. Chromosome spreads of HCMV-pp150/pUL21a-RXLmut infected cells were subjected to Giemsa staining and compared to equally prepared material of HCMV-UL21a-RXLmut infected cells from 1 to 4 days post infection. Representative images are shown.
(TIF)

S8 Fig RXL double mutant infection results in rapid loss of viable cells. S phase fibroblasts were infected with HCMV-WT or the indicated RXL mutants. Cells were harvested at regular intervals. Immediately after harvest, cells were subjected to propidium iodide (PI) staining and flow cytometry. Forward scatter (FSC) and sideward scatter (SSC) were used to define a region (R1) that excludes cellular debris from analysis (upper panel). PI fluorescence was analyzed to determine the percentage of viable, PI excluding cells (R2) in the parental region R1. Events originating from R2 are highlighted in red. The experiment was performed twice in triplicates with similar results. Representative dot plots are shown.
(TIF)
