# Supplementary figures and images for: Synthetic lethal mutations in the cyclin A interface of human cytomegalovirus
Source: PLoS Pathog. 2017 Jan 27;13(1):e1006193. doi: 10.1371/journal.ppat.1006193 (PMC5298330; doi:10.1371/journal.ppat.1006193)

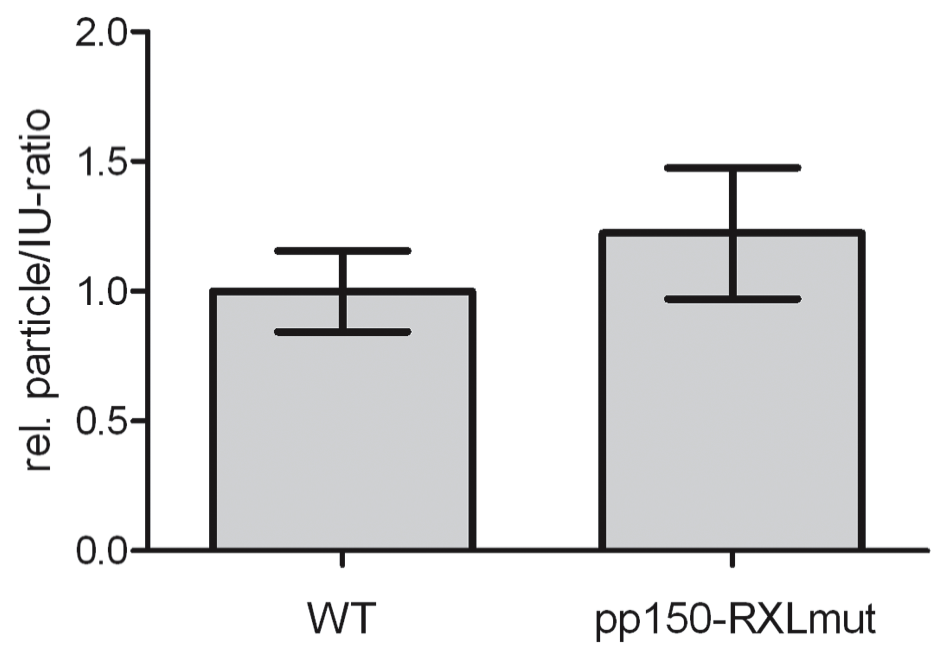

Supplement: S1 Fig — Virus stocks of HCMV-WT and HCMV-pp150-RXLmut were titrated by determining the concentration of infectious, IE1/IE2 protein forming units (IU). In addition, virion DNA was isolated from virus stocks and quantified by real-time PCR. The histogram shows relative particle to IU ratios of HCMV-WT and pp150-RXLmut, with WT set to 1.0. Data represent the means and standard deviations of technical triplicates. (TIF) [file ppat.1006193.s001.tif]

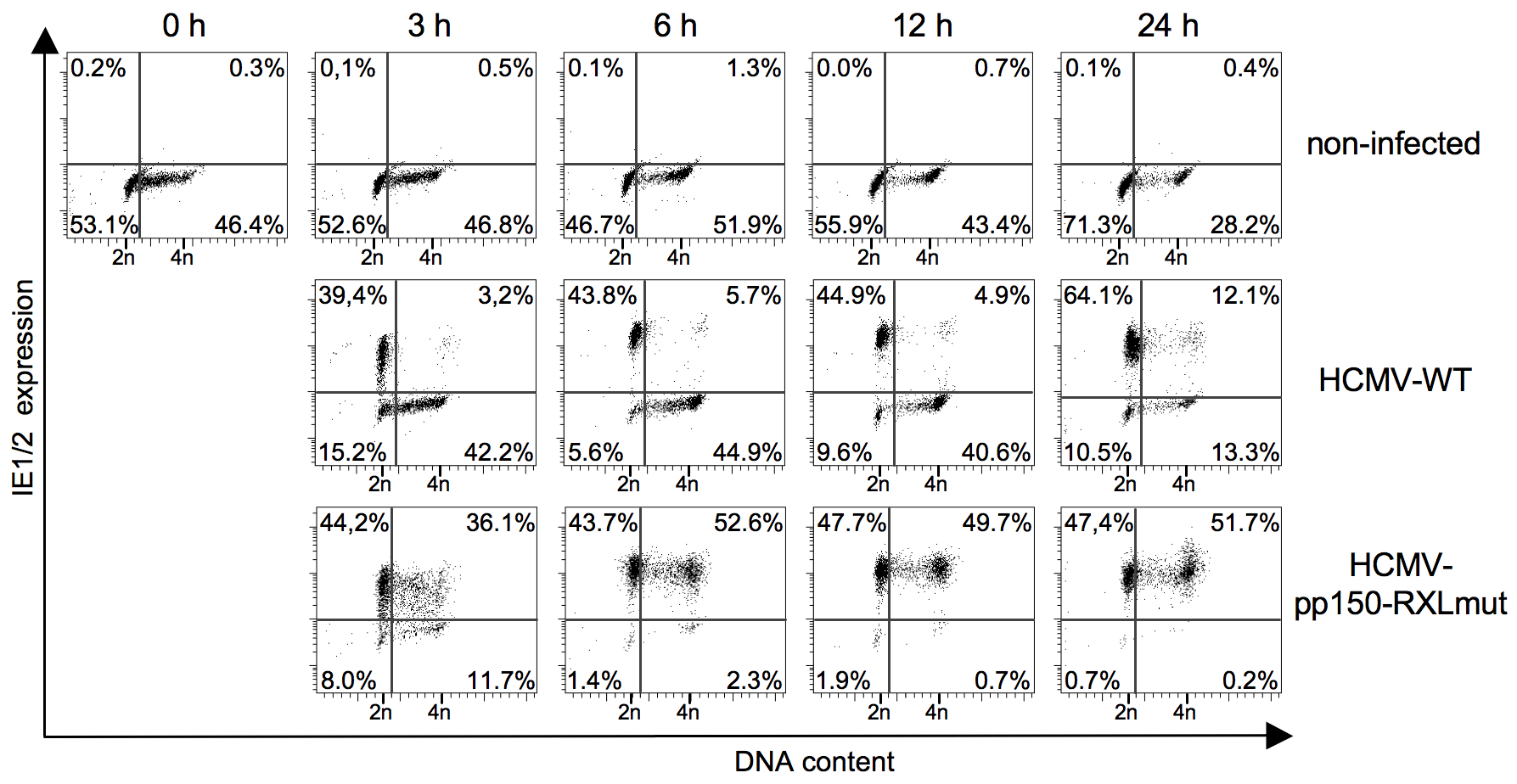

Supplement: S2 Fig — Embryonic lung fibroblasts were partially synchronized in early S phase and infected with pp150-WT or pp150-RXL mutant HCMV. Cellular DNA content and expression of immediate early gene products IE1 and IE2 were analyzed during the first 24 h post infection by flow cytometry (n = haploid number of chromosomes). (TIF) [file ppat.1006193.s002.tif]

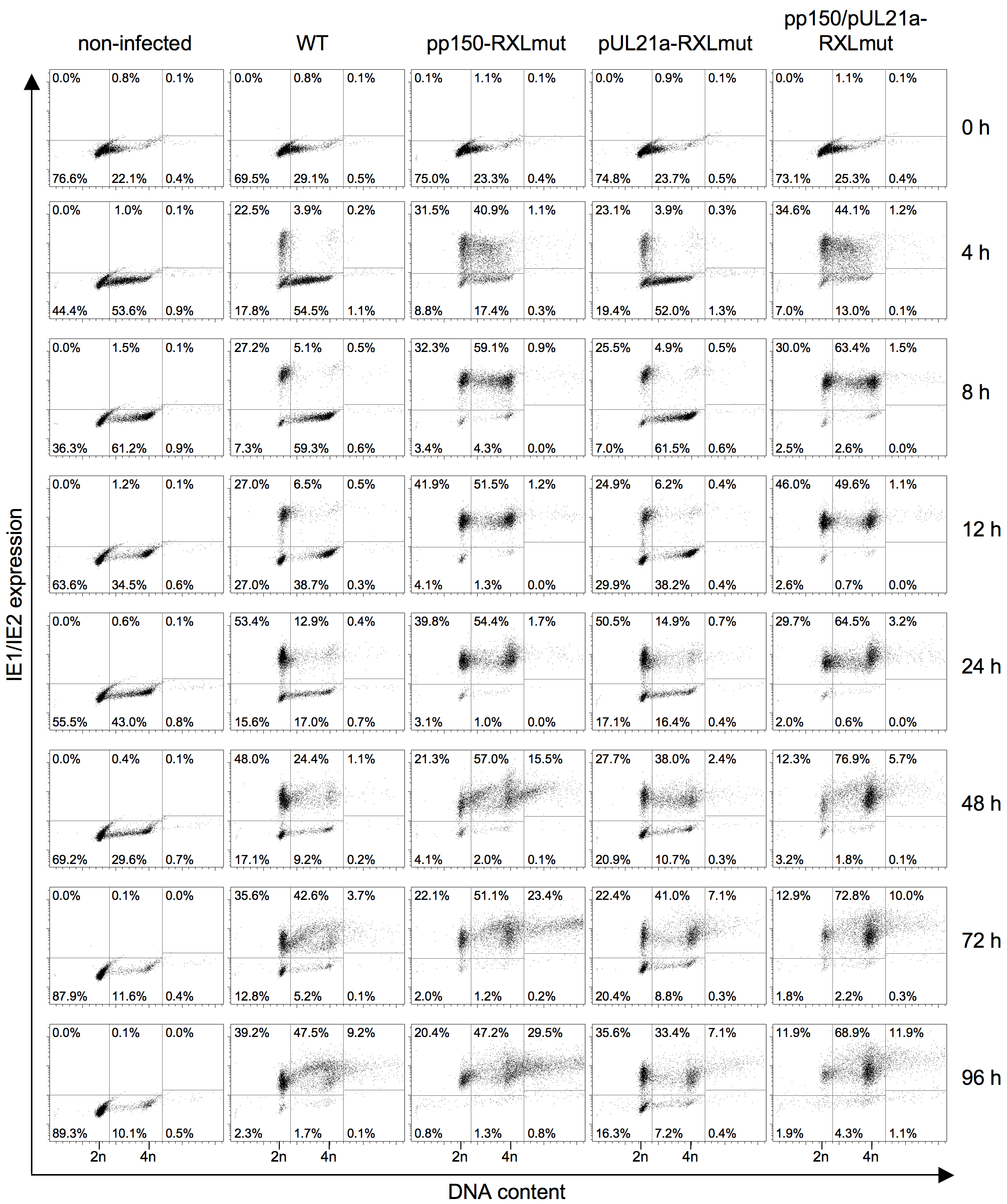

Supplement: S3 Fig — Partially synchronized fibroblasts were infected near the G1/S transition with the indicated HCMV variants. Major immediate early (IE) gene expression and DNA content were analyzed by flow cytometry on a daily basis. Shown are dot plots where the cellular events are divided into 6 subpopulations. Upper left region: IE+/DNA content = 2n; lower left region: IE-/DNA content = 2n; upper middle region: IE+/DNA content >2n and ≤4n; lower middle region: IE-/DNA content >2n and ≤4n; upper right region: IE+/DNA content >4n; lower right region: IE-/DNA content >4n (n = haploid number of chromosomes). A DNA content >4n indicates viral DNA replication of G2 arrested cells. (TIF) [file ppat.1006193.s003.tif]

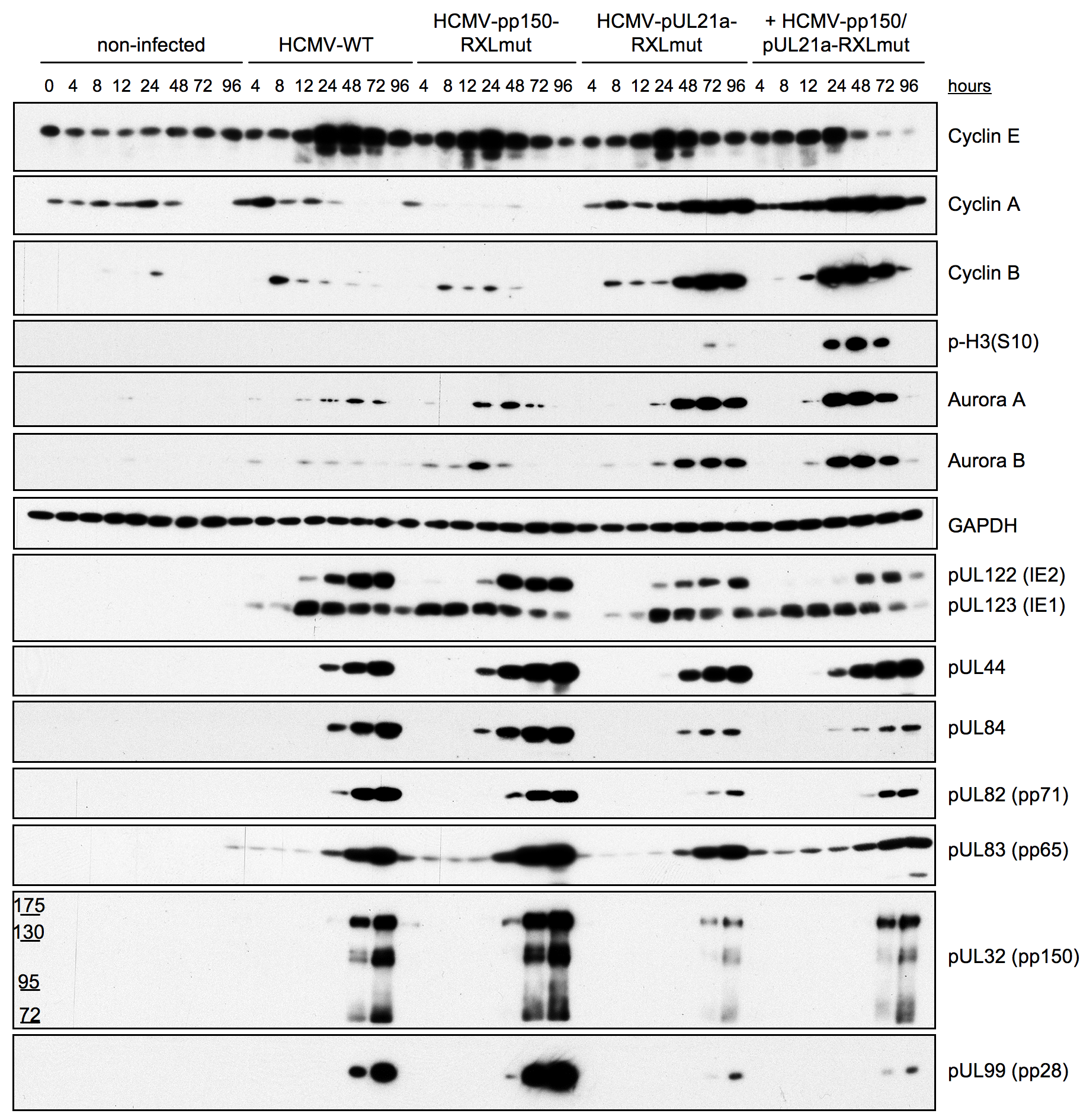

Supplement: S4 Fig — G1/S fibroblasts were infected with HCMV-WT, the indicated RXL mutants or left uninfected. Whole cell lysates were prepared from 0 to 96 h post infection and analyzed by immunoblotting for protein expression of cyclins, mitotic kinases and selected immediate early, early and late gene products. In addition, histone H3-serine 10 phosphorylation, pH3(ser10), was analyzed. The conditions used for immunoblot detection of pH3(ser10) were not sensitive enough to allow a comparison of pH3(ser10) levels in non-infected and HCMV-WT infected cells. Equal protein amounts were loaded, which was controlled by analysis of GAPDH expression. (TIF) [file ppat.1006193.s004.tif]

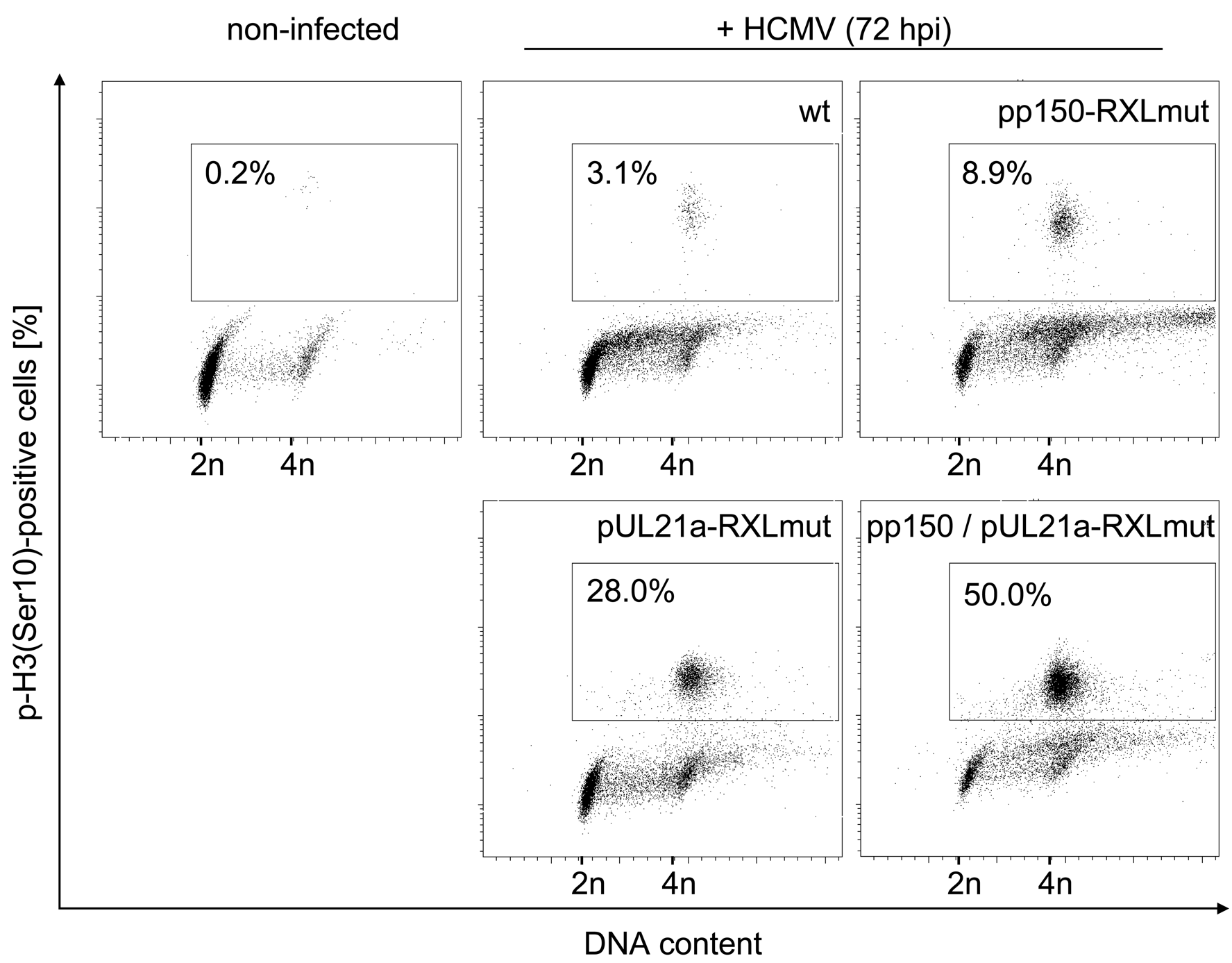

Supplement: S5 Fig — Fibroblasts were synchronized and infected as described above. After harvest (here: at 72 h), cells were stained with propidium iodide and monoclonal antibodies against IE1/IE2 and pH3(ser10). The percentage of mitotic, pH3(ser10) positive cells was assessed by flow cytometry. Only the fraction of IE1/IE2-positive cells was included in the analysis. (TIF) [file ppat.1006193.s005.tif]

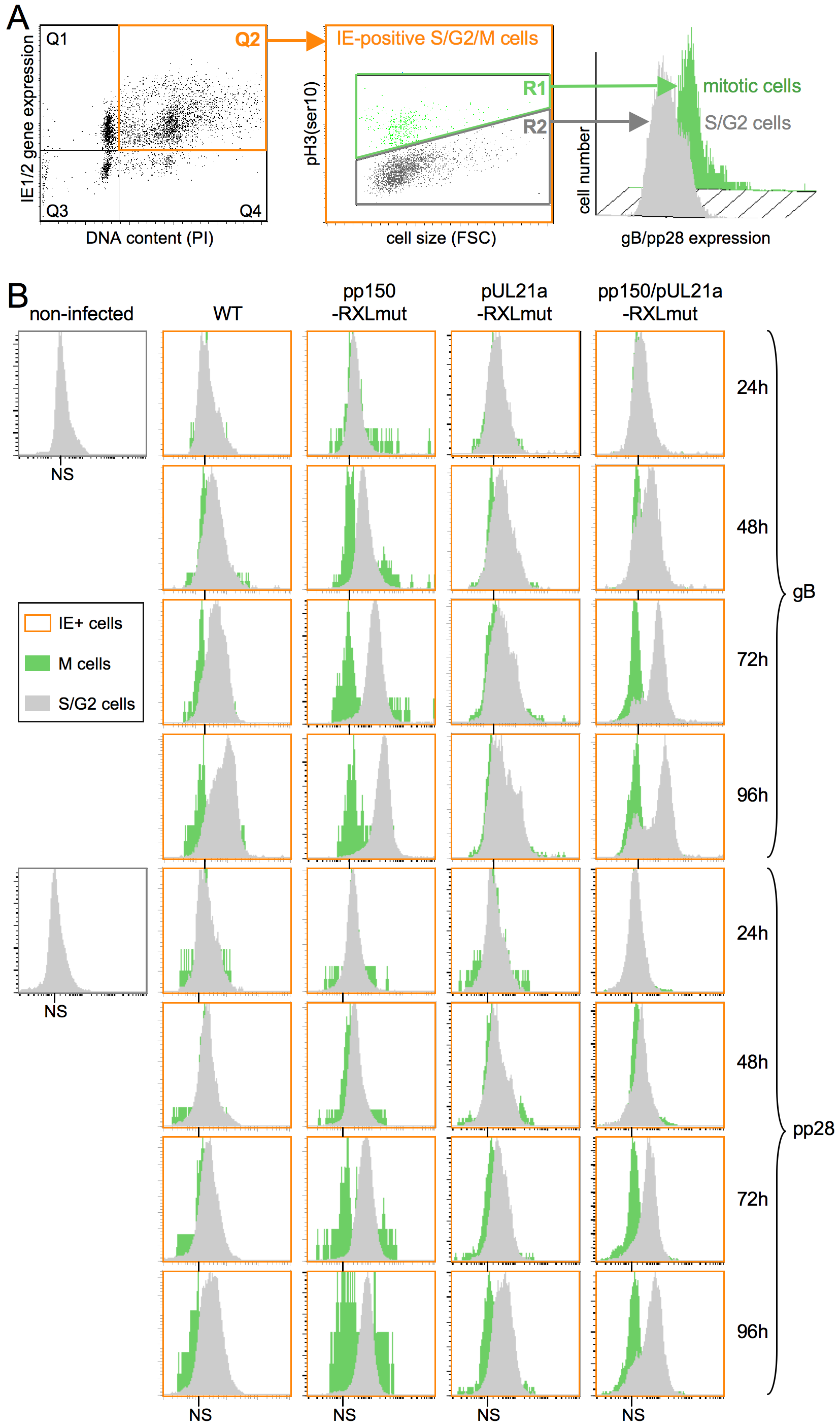

Supplement: S6 Fig — Fibroblasts were infected in early S phase with HCMV-WT or the indicated RXL mutants. Cellular DNA content, mitotic marker pH3(ser10) and expression of viral immediate early (IE1/2), early (gB) and late (pp28) proteins were analyzed by flow cytometry at the indicated time points. (A) To test how efficiently the HCMV replication cycle proceeds in S/G2 versus M phase, a gating strategy was designed where the quadrant of IE-positive S/G2/M cells (Q2) was subdivided into a pH3(ser10)-positive mitotic population (R1) and a pH3(ser10)-negative S/G2 population (R2). Both populations were compared with respect to gB and pp28 protein expression. (B) Shown are histogram overlays of IE-positive mitotic and S/G2 populations. Non-infected S/G2 cells were analyzed to control for non-specific (NS) background staining. (TIF) [file ppat.1006193.s006.tif]

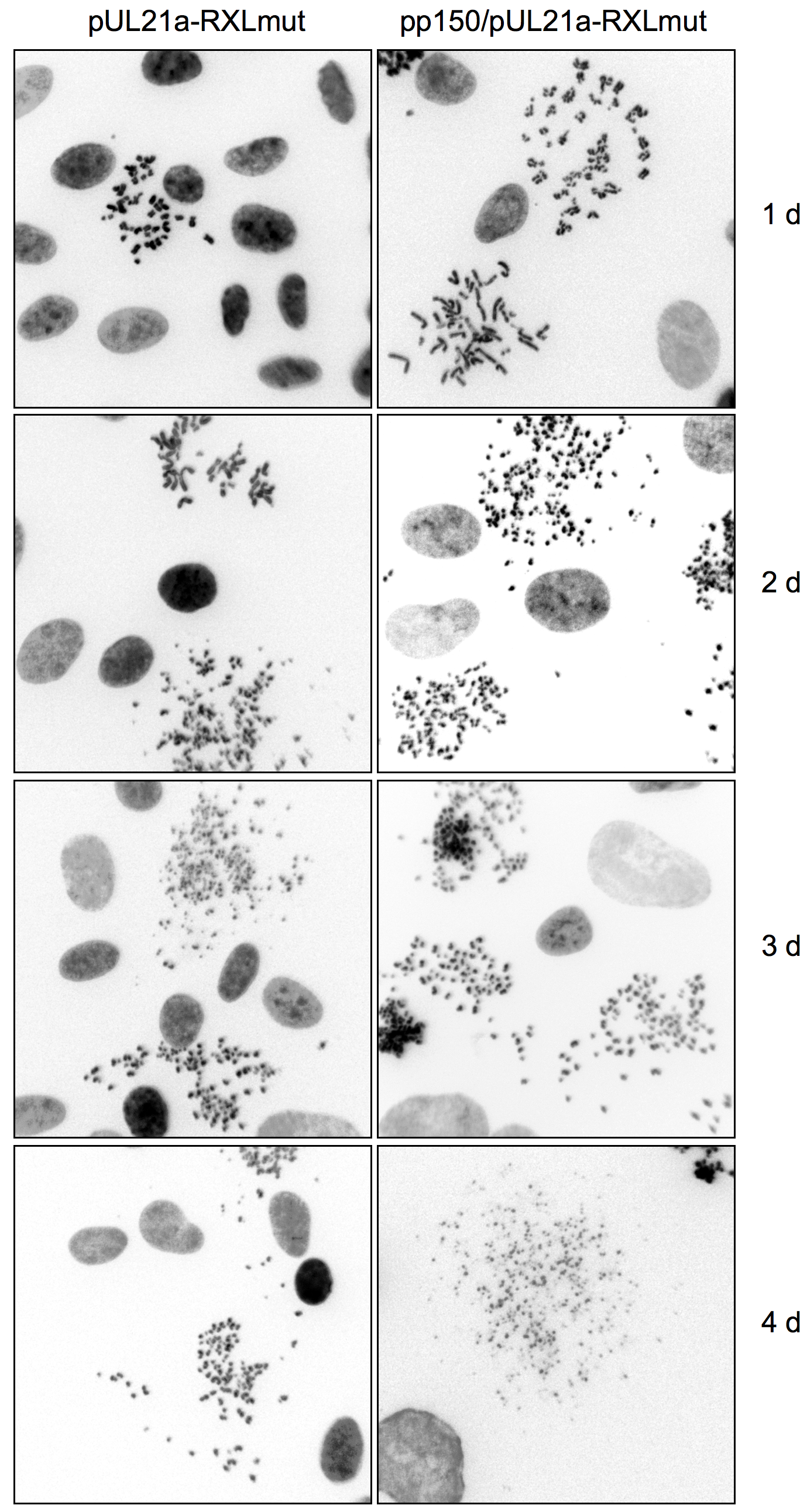

Supplement: S7 Fig — Chromosome spreads of HCMV-pp150/pUL21a-RXLmut infected cells were subjected to Giemsa staining and compared to equally prepared material of HCMV-UL21a-RXLmut infected cells from 1 to 4 days post infection. Representative images are shown. (TIF) [file ppat.1006193.s007.tif]

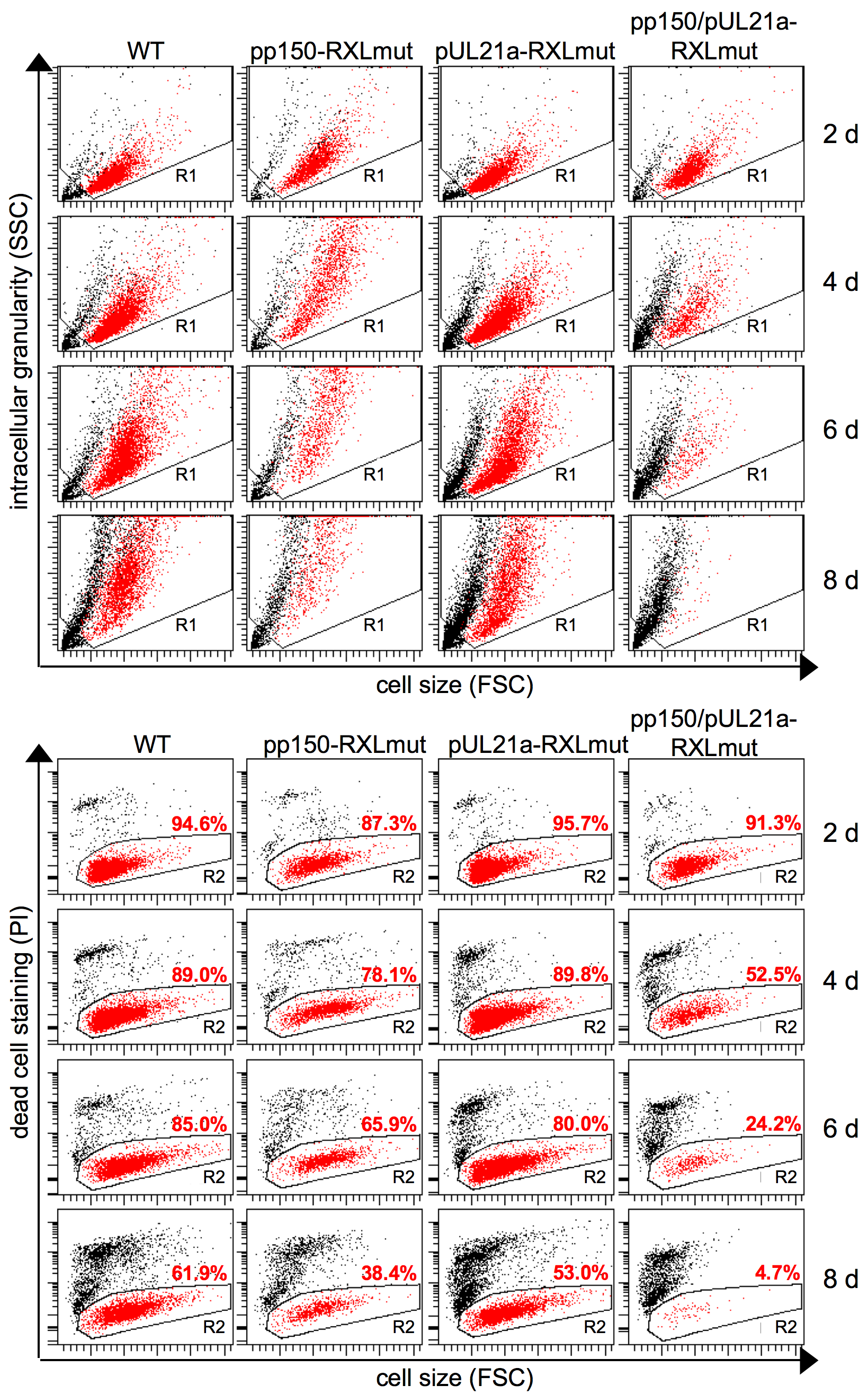

Supplement: S8 Fig — S phase fibroblasts were infected with HCMV-WT or the indicated RXL mutants. Cells were harvested at regular intervals. Immediately after harvest, cells were subjected to propidium iodide (PI) staining and flow cytometry. Forward scatter (FSC) and sideward scatter (SSC) were used to define a region (R1) that excludes cellular debris from analysis (upper panel). PI fluorescence was analyzed to determine the percentage of viable, PI excluding cells (R2) in the parental region R1. Events originating from R2 are highlighted in red. The experiment was performed twice in triplicates with similar results. Representative dot plots are shown. (TIF) [file ppat.1006193.s008.tif]
